# Supplementary material for: Ferroptosis-Linked Six-Gene Panel Enables Machine Learning-Assisted Diagnosis and Therapeutic Guidance in Lung Adenocarcinoma
Source: Biology (Basel). 2025 Sep 17;14(9):1280. doi: 10.3390/biology14091280 (PMC12467038; doi:10.3390/biology14091280)
Supplement: Supplementary file 1 [file biology-14-01280-s001.zip › biology-3825212-Supplementary Figures.pdf]

Supplementary Figures

Ferroptosis-linked six-gene panel enables machine learning-assisted diagnosis and therapeutic guidance in lung adenocarcinoma

* Corresponding Author: Faris Alrumaihi, Department of Medical Laboratories, College of Applied Medical Sciences, Qassim University, Buraydah 51452, Saudi Arabia, Email: f_alrumaihi@qu.edu.sa

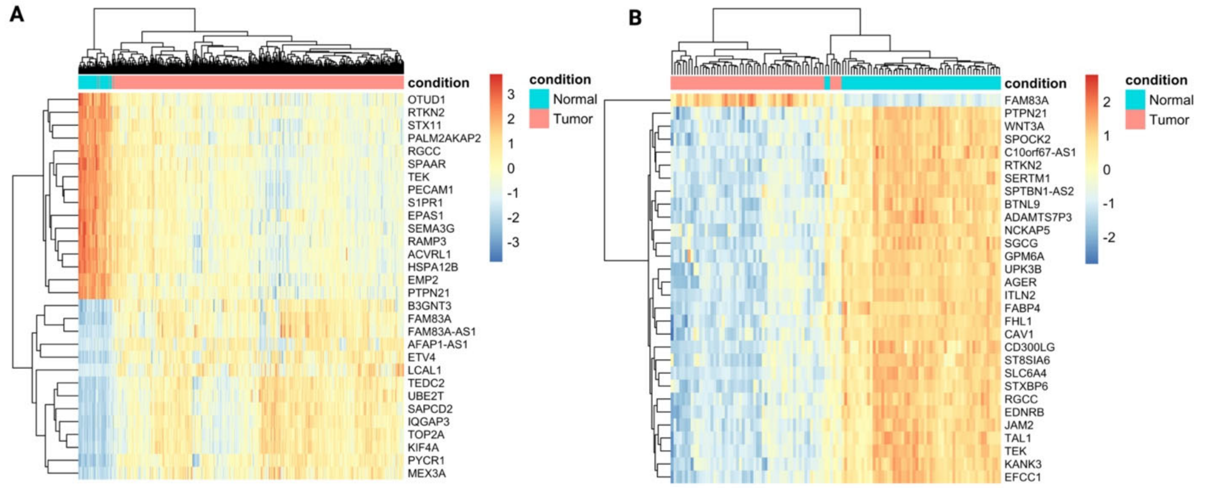

Supplementary Figure S1: (A) Heatmap of the top 30 most significant DEGs from the unpaired analysis, showing clear segregation of tumour and normal samples (B) Heatmap of the top 30 DEGs from the paired analysis, highlighting consistent within-patient tumour-normal differences.

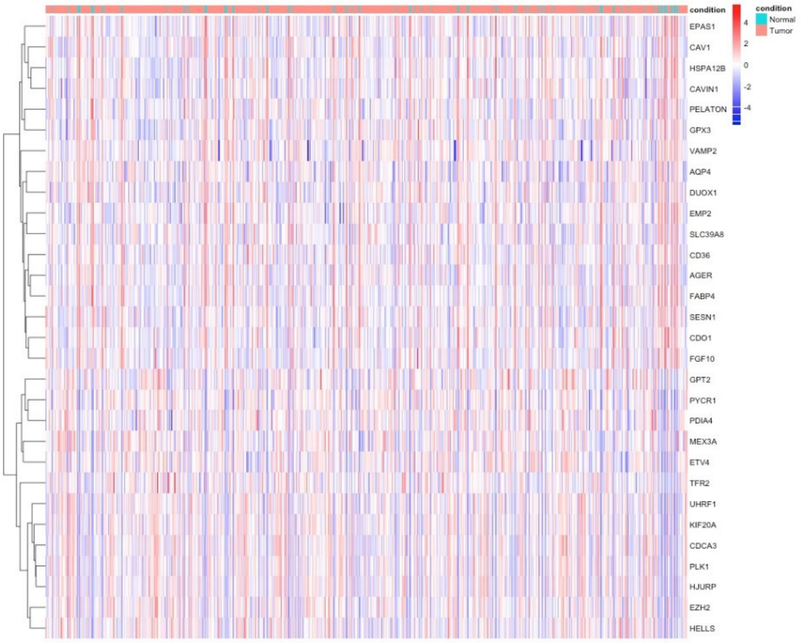

Supplementary Figure S2: Heatmap showing expression patterns of the top 30 ferroptosis-related genes ranked by adjusted P-value. Rows represent genes, columns represent samples. Tumour samples are indicated in red, and normal samples in blue. Expression values are scaled by row.

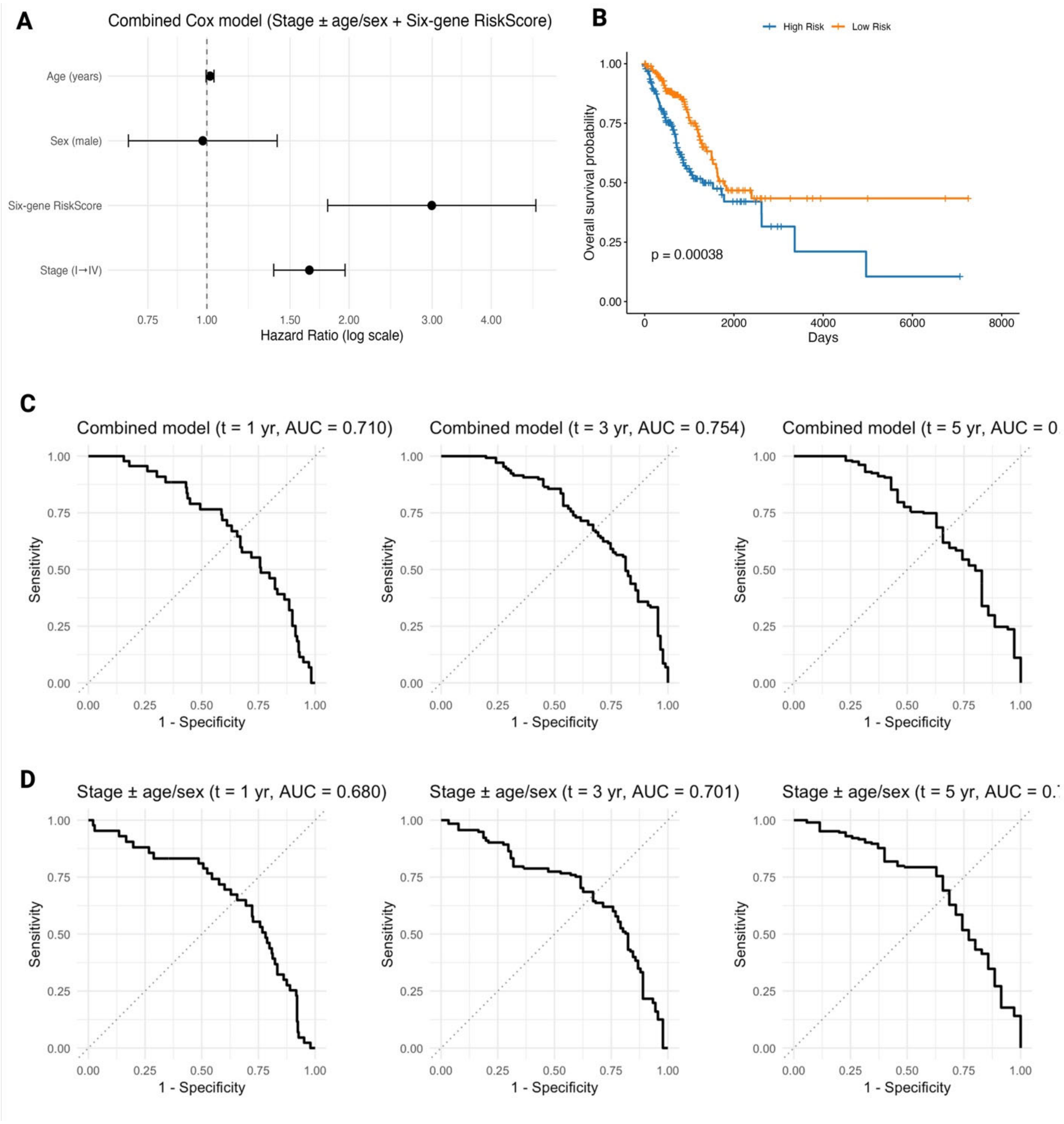

Supplementary Figure S3. Prognostic performance of the six-gene ferroptosis signature in TCGA-LUAD. (A) Forest plot from multivariable Cox regression including AJCC stage, age, sex, and six-gene RiskScore. Both stage and RiskScore were independently associated with overall survival. (B) Kaplan–Meier survival curves stratified by median RiskScore (log-rank p = 3.8×10^{-4}). (C) Time-dependent ROC curves at 1, 3, and 5 years for the combined model (stage + RiskScore). (D) Time-dependent ROC curves at 1, 3, and 5 years for stage ± age/sex alone.

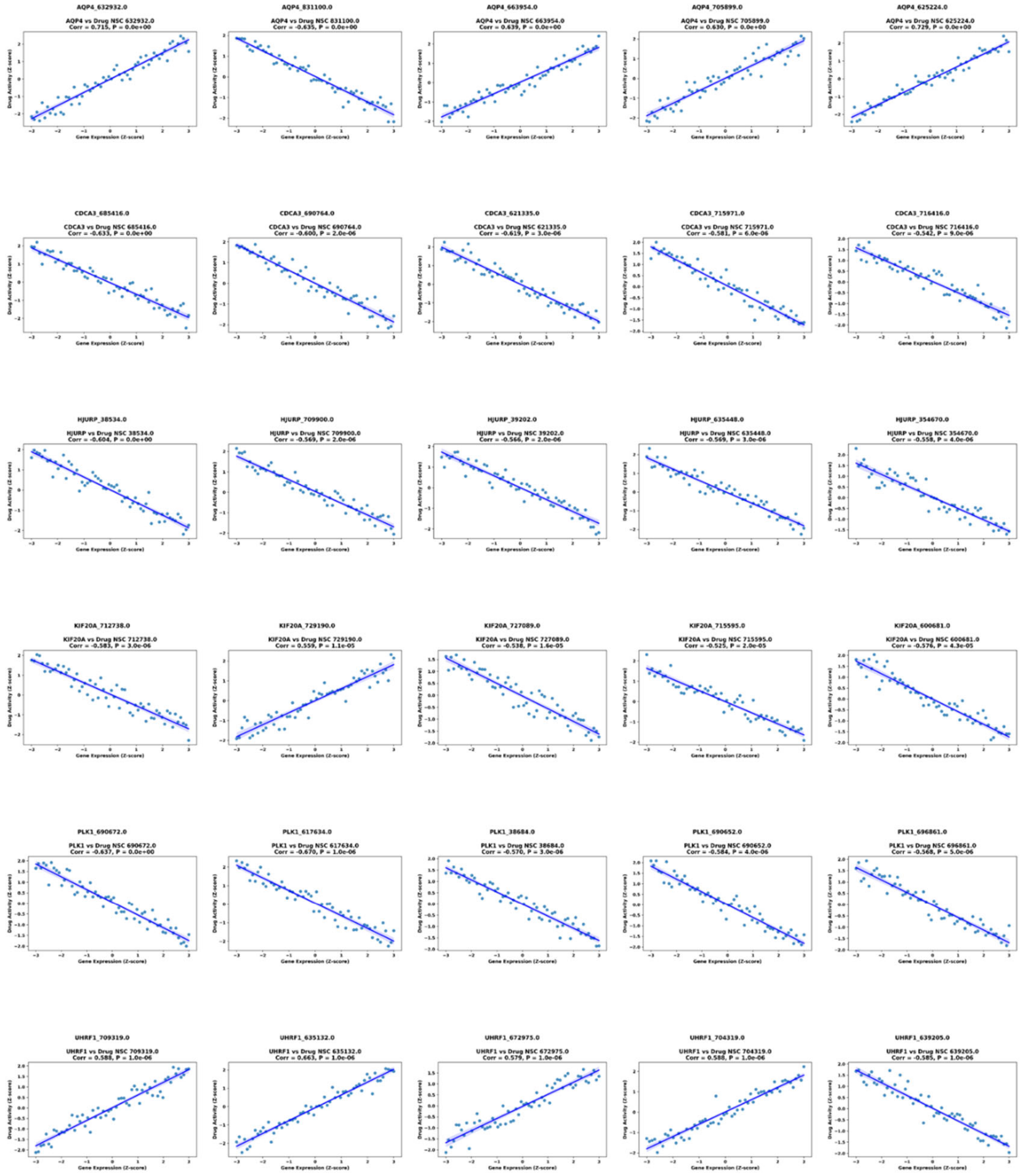

Supplementary Figure S4. Associations between prognostic gene expression and drug sensitivity across the NCI-60 cancer cell line panel. Scatter plots depict Pearson correlations between the expression of each gene in the six-gene panel and compound activity (Z-scores) obtained from the CellMiner database. Each panel shows a representative gene–drug pair, annotated with the NSC identifier and correlation coefficient. Negative correlations indicate increased drug sensitivity with higher gene expression, whereas positive correlations imply reduced sensitivity or potential resistance. While these associations highlight candidate therapeutic vulnerabilities linked to proliferation-associated gene expression, they are derived from a pan-cancer dataset and should be considered exploratory pending validation in LUAD-specific models. For readability, the complete ranked associations are provided in Supplementary Table S15 (top five per gene, r and FDR-adjusted P).
